# When Operating on Dead People Saves Lives: Benefits of Surgical Organ Donor Intensivists

**DOI:** 10.1155/2015/608673

**Published:** 2015-05-11

**Authors:** Kristin Long, Cynthia Talley, Rebecca B. Yarrison, Andrew Bernard

**Affiliations:** ^1^University of Kentucky Department of Surgery, Lexington, KY 40536, USA; ^2^Division of Trauma & Acute Care Surgery, Department of Surgery, University of Kentucky, Lexington, KY 40536, USA; ^3^University of Kentucky Department of Medicine, Bioethics, Lexington, KY 40536, USA

## Abstract

Solid organ transplantation has emerged as a life-saving treatment for many patients suffering from end-stage organ failure. Organs have been successfully recovered after a variety of aggressive interventions. We propose that decompressive laparotomy, when clinically indicated, should be considered in the aggressive resuscitation of potential organ donors. A thorough literature review examining aggressive interventions on potential organ donors was conducted after experience with a unique case at this institution. Articles were reviewed for the types of interventions performed as well as the time frame in relation to organ donation. In our case, several ethical issues were raised when considering decompressive laparotomy in a patient pronounced dead by neurologic criteria. We propose that having a surgical intensivist involved in the management of potential donors will further increase the salvage rate, as more invasive resuscitation options are possible.

## 1. Introduction

Solid organ transplantation has emerged as a life-saving treatment for many patients suffering from end-stage organ failure. While the volume of patients on the transplant waiting list continues to rise each year, the number of organs donated falls far short of the number needed. Currently, there are over 131,000 patients waiting for a solid organ transplant, and only 11,800 donors were recovered in 2013 [1]. Aggressive management protocols for potential organ donors have been implemented in hospitals across the country, resulting in an increased number of successful procurements [2]. Dedicated organ donor intensivists have proven useful in guiding resuscitation of potential donors, and organs have been successfully recovered after aggressive interventions such as extracorporeal membrane oxygenation (ECMO), damage control surgery, and decompressive laparotomy [3–6]. Here, we present a case of a decompressive laparotomy performed for abdominal compartment syndrome (ACS) in potential organ donor already declared dead by neurological criteria whose next of kin had already consented to donation. The procedure led to successful organ recovery and demonstrates the benefit of surgical intensivist involvement in resuscitation and management of potential of organ donors.

## 2. Case Report

A 52-year-old male was admitted with a severe intracranial abscess and progressed to a diagnosis of death by neurological criteria. The local organ procurement organization (OPO) evaluated the patient for potential organ donation and obtained informed consent from the next of kin for organ donation and potential resuscitative interventions. Aggressive resuscitation was continued. Our center uses an organ donor intensivist (ODI) consult service model with medical, anesthesia, and surgical intensivists sharing ODI call coverage. The patient required vasopressors and intravenous fluid resuscitation. Several hours into his resuscitation, his abdomen became increasingly distended and firm. Peak airway pressures were elevated to 34 mmHg, and a bladder pressure obtained after administration of paralytic medication was elevated at 31 mmHg. Concerned for abdominal compartment syndrome, the ODI contacted the acute care surgical service for discussion of decompressive laparotomy. The attending surgeon on the acute care surgery service was a fellow ODI. Approximately 90 minutes after this diagnosis, the patient was taken to operating room where decompressive laparotomy was performed and a temporary abdominal closure device was placed. Peak airway pressures improved after operative intervention, and the patient returned to the intensive care unit for continued resuscitation. Later the next day, liver and kidneys were successfully procured for transplant and the liver was successfully transplanted. The recipient is reportedly doing well.

## 3. Discussion

Aggressive resuscitation of potential donors has increased the number of organs available for transplantation. Use of intensivists and structured resuscitation protocols has contributed to the improved recovery efforts [2]. We propose that having a surgical intensivist involved in the management of potential donors will further increase the number of organs salvaged, as more invasive resuscitation options are available. These include damage control laparotomy for hemorrhaging patients, decompressive laparotomy for patients developing abdominal compartment syndrome during their resuscitation, and even ECMO for patients with cardiopulmonary compromise. Operative procedures in the donor resuscitative phase also allow for direct tissue biopsy of liver and kidneys, which may facilitate organ placement.

With all procedures performed in the management of potential organ donors, ethical considerations are particularly important and sensitive. The key ethical issues are related to financial responsibility for the procedures, proper informed consent, and the ethical appropriateness of aggressive, invasive procedures in an already-dead donor [7].

1. Because the next of kin had already consented to organ donation, the OPO bears the costs of the procedure. Neither the donor's insurance company nor the donor's estate bears any of the associated costs.
2. The informed consent process with the next of kin included an explanation of the possible procedures used to optimize organ donation potential, including invasive surgical procedures if necessary. This encompasses the decompressive laparotomy, but this procedure was not anticipated and therefore not specifically discussed with the donor's next of kin. The next of kin was not available prior to the procedure for additional discussions. When possible, informing the next of kin of this additional operative intervention outside of procurement is best in caring for the donor and respecting the family.
3. In this case, the decompressive laparotomy can be justified because the potential benefit of organ preservation for donation outweighs the risks associated with the laparotomy. Since the potential donor is already dead, he could not be harmed in the sense of pain, morbidity, or mortality. Concerns about causing distress to the family and respecting the body's dignity are addressed by seeking the consent of the family and treating the body with respect, which includes only performing the procedures necessary and performing them with the same attention and consideration that we would do with a living patient. But the fact that the potential donor cannot suffer pain, morbidity, or mortality does not mean that any and all efforts would be justified. Exactly where the line should be drawn would depend on the details of a particular case: how strongly the potential donor and his next of kin wished for donation and what they are willing to consent to, how likely it is that the organs will be useable after the procedure, how aggressive and invasive the procedure is, whether the use of the operating room or other resources would make those resources unavailable to a living patient who needs them, and other such considerations.

Medical and anesthesia intensivists are extremely beneficial in optimizing most resuscitation parameters but are limited in the ability to perform certain invasive procedures like laparotomy. This highlights the benefit of having surgical involvement in the organ donor management program. Acute care surgeons, specializing in trauma, emergency surgery, and critical care, are ideally suited to this supportive role for the organ donor management program in donor centers. Many potential donors present as a result of devastating traumatic injuries and represent a patient population extremely familiar to trauma and acute care surgeons. Isolated case reports detail success in using damage control laparotomy and decompressive laparotomy specifically in attempts to salvage organs; however, in our literature search, only one report of each was noted [3, 4].

In summary, this case represents an important example of aggressive treatment and resuscitation resulting in successful procurement of multiple organs for transplant. With the advent of expanded-donor criteria and the increasing urgency in need for organs, surgical salvage procedures should be considered in all potential organ donors. Decisions in these cases should be guided by a multidisciplinary team including organ procurement organizations and surgical intensivists.

## References

[1] United States Department of Health and Human Services Organ Procurement and Transplantation Network http://optn.transplant.hrsa.gov/latestdata/rptdata.asp.

[2] Singbartl K., Murugan R., Kaynar A. M. (2011). Intensivist-led management of brain-dead donors is associated with an increase in organ recovery for transplantation. *The American Journal of Transplantation*.

[3] Rollins M. D., Deamorim-Filho J., Scaife E. R., Hubbard A., Barnhart D. C. (2013). Decompressive laparotomy for abdominal compartment syndrome in children on ECMO: effect on Support and Survival. *Journal of Pediatric Surgery*.

[4] Ahmed N., Cheng-Robles D. (2006). Damage control surgery prior to organ harvesting. *Journal of Trauma*.

[5] Zuckerman J. M., Singh R. P., Farney A. C., Rogers J., Hines M. H., Stratta R. J. (2009). Successful kidney transplantation from a donation after cardiac death donor with acute renal failure and bowel infarction using extracorporeal support. *Transplant International*.

[6] Manning B. M., Arrillaga A., Miller R. S., Kopelman T. (2001). Abdominal decompression prior to organ harvesting: case report. *The Journal of Trauma-Injury Infection & Critical Care*.

[7] Hope W. W., Jacobs D. G., Stell L. K., Sing R. F. (2007). Comment on ‘damage control surgery prior to organ harvesting’. *The Journal of Trauma*.

